# Co-administration of Anti microRNA-124 and -137 Oligonucleotides Prevents Hippocampal Neural Stem Cell Loss Upon Non-convulsive Seizures

**DOI:** 10.3389/fnmol.2019.00031

**Published:** 2019-02-19

**Authors:** Pascal Bielefeld, Marijn Schouten, Guido M. Meijer, Marit J. Breuk, Karlijne Geijtenbeek, Sedef Karayel, Alisa Tiaglik, Anna H. Vuuregge, Ruth A.L. Willems, Diede Witkamp, Paul J. Lucassen, Juan M. Encinas, Carlos P. Fitzsimons

**Affiliations:** ^1^Neuroscience Program, Swammerdam Institute for Life Sciences, Faculty of Sciences, University of Amsterdam, Amsterdam, Netherlands; ^2^Achucarro Basque Center for Neuroscience, Bizkaia Science and Technology Park, Zamudio, Spain; ^3^Ikerbasque Foundation, Bilbao, Spain; ^4^University of the Basque Country (UPV/EHU), Leioa, Spain

**Keywords:** non-convulsive seizures, kainic acid, adult hippocampal neurogenesis, neural stem cell fate, microRNA

## Abstract

Convulsive seizures promote adult hippocampal neurogenesis (AHN) through a transient activation of neural stem/progenitor cells (NSPCs) in the subgranular zone (SGZ) of the dentate gyrus (DG). However, in a significant population of epilepsy patients, non-convulsive seizures (ncSZ) are observed. The response of NSPCs to non-convulsive seizure induction has not been characterized before. We here studied first the short-term effects of controlled seizure induction on NSPCs fate and identity. We induced seizures of controlled intensity by intrahippocampally injecting increasing doses of the chemoconvulsant kainic acid (KA) and analyzed their effect on subdural EEG recordings, hippocampal structure, NSPC proliferation and the number and location of immature neurons shortly after seizure onset. After establishing a KA dose that elicits ncSZ, we then analyzed the effects of ncSZ on NSPC proliferation and NSC identity in the hippocampus. ncSZ specifically triggered neuroblast proliferation, but did not induce proliferation of NSPCs in the SGZ, 3 days post seizure onset. However, ncSZ induced significant changes in NSPC composition in the hippocampus, including the generation of reactive NSCs. Interestingly, intrahippocampal injection of a combination of two anti microRNA oligonucleotides targeting microRNA-124 and -137 normalized neuroblast proliferation and prevented NSC loss in the DG upon ncSZ. Our results show for the first time that ncSZ induce significant changes in neuroblast proliferation and NSC composition. Simultaneous antagonism of both microRNA-124 and -137 rescued seizure-induced alterations in NSPC, supporting their coordinated action in the regulation of NSC fate and proliferation and their potential for future seizure therapies.

## Introduction

Convulsive seizures (cSZ) affect the hippocampus and promote adult hippocampal neurogenesis (AHN). A subset of adult-generated granule cells born after cSZ develop and integrate aberrantly in the hippocampus and have been implicated in circuit disinhibition, continued seizure formation, and epileptogenesis (Parent et al., 1997; Pun et al., 2012; Cho et al., 2015; Singh et al., 2015). Furthermore, the excessive activation of hippocampal Neural Stem/Progenitor Cells (NSPCs) that occurs shortly after seizure onset triggers their aberrant proliferation and may thereby deplete the neurogenic NSPC pool and limit AHN (Encinas et al., 2011; Sierra et al., 2015), contributing to some of the cognitive deficits that often accompany epilepsy (Hattiangady and Shetty, 2008; Cho et al., 2015).

Previous studies have suggested that the NSPC response to seizure stimulation may depend on seizure intensity, leading to differences in pathological outcome (Mohapel et al., 2004; Hung et al., 2012; Sierra et al., 2015; Uemori et al., 2017) (reviewed in Bielefeld et al., 2014). The induction of cSZ by kainic acid (KA) activates quiescent, radial glia-like NSCs in the hippocampus, promotes their proliferation, alters cell-fate decisions and results in a shift from a mainly neurogenic toward a strongly astrogenic fate (Lugert et al., 2010; Sierra et al., 2015). Importantly, a significant population of epilepsy patients never experience cSZ, but often suffer from milder, non-convulsive seizures (ncSZ) (Labovitz et al., 2001; Korff and Nordli, 2007; Rosenow et al., 2007), which have also been appreciated in rodent models (Kienzler-Norwood et al., 2017; Avdic et al., 2018). However, the effects of ncSZ on hippocampal NSPC proliferation and cell-fate decisions remains poorly characterized.

Neural stem cell fate choices depend on the expression of specific sets of co-regulated genes, that are often controlled by lineage-specific transcription factors and microRNAs (miRNAs) (Encinas and Fitzsimons, 2017; Llorens-Bobadilla and Martin-Villalba, 2017). miRNAs are short single-stranded non-coding RNAs, that post-transcriptionally repress target mRNAs through imperfect RNA-RNA binding (Bartel, 2004; Pasquinelli, 2012; Wilczynska and Bushell, 2015). miRNAs can act synergistically on the same gene targets, or on targets involved in the same biological process, creating an additional layer of regulatory complexity (Barca-Mayo and De Pietri Tonelli, 2014). Highly coordinated synergistic miRNA actions control neuronal fate in adult hippocampal NSPCs (Schouten et al., 2015; Pons-Espinal et al., 2017). The synergistic action of multiple miRNAs in NSPCs provides a possible mechanism that may render target genes more sensitive to relatively small changes in the level of individual miRNAs, thereby effectively reducing the number of biologically relevant targets (Barca-Mayo and De Pietri Tonelli, 2014; Schouten et al., 2015).

Here, we set out to study the effect of ncSZ on hippocampal NSPC proliferation and fate decisions and its regulation by the synergistic action of miR-124 and -137, which are upregulated in the mouse dentate gyrus (DG) shortly after KA-induced seizures (Schouten et al., 2015, 2016). To further characterize the synergistic role of miRNA-124 and -137 in NSPC *in vivo*, we used synthetic antimicroRNA oligonucleotides (AMOs) (Weiler et al., 2006; Lennox et al., 2013) which bind specifically to miRNAs and block their action, thereby allowing a loss-of-function study of miRNA activity (Velu and Grimes, 2012; Li and Rana, 2014).

## Results

### Seizure Intensity Conditions the Cellular Response in the Hippocampus

**FIGURE 1:**
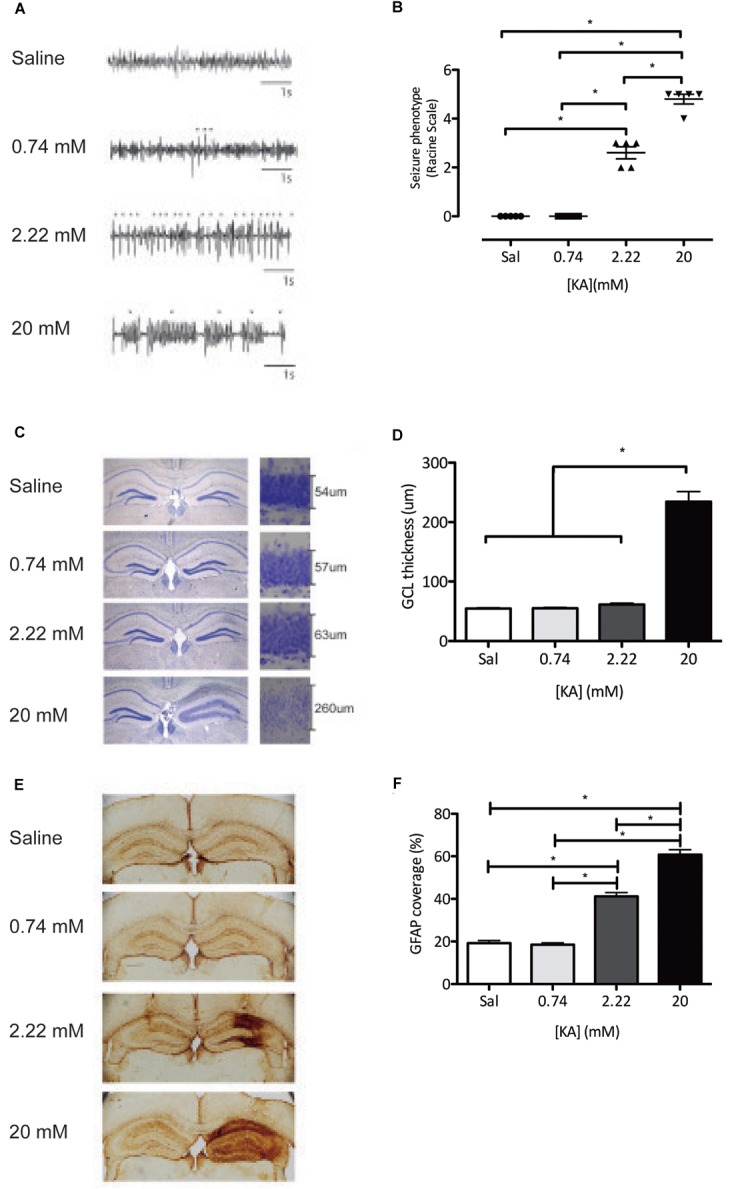
Characterization of the intrahippocampal dose-dependent KA model. **(A)** EEG recordings during the first 4 h of status epilepticus show clear divergent patterns dependent on the KA dose, varying from single spikes(^∗^) (0.74 mM) and repetitive single spikes (2.22 mM), to repetitive spike-bursts (∇) (20 mM). **(B)** Classification of behavioral seizures during the first 4 h of status epilepticus assessed using the Racine scale. **(C)** A Nissl staining shows dispersion of the granule cell layer. **(D)** Quantification of the granule dispersion 28 days after KA administration. **(E)** Immunohistochemistry against GFAP reveals KA dose-dependent induction of astrogliosis. **(F)** Quantification of GFAP coverage of the total hippocampus 28 days after KA administration. ^∗^*P* < 0.05.

We first studied the effects on seizure activity recorded by subdural EEG of increasing intrahippocampal KA doses, within a range of previously established doses that induce interictal spiking (0.74 mM) (Sierra et al., 2015; Bielefeld et al., 2017), low-grade seizures (2.22 mM) (Bielefeld et al., 2017), and tonic-clonic seizures (20 mM) (Bouilleret et al., 1999; Sierra et al., 2015). We then analyzed granule cell dispersion and astrogliosis in the hippocampus. Injection of 0.74 mM KA elicited neuronal discharges in the form of single spikes compatible with epileptiform activity but no seizure activity was detected in subdural EEG recordings, as compared to saline injections (Figure 1A). Two point twenty two millimeter KA was the first dose to evoke seizures detectable by subdural EEG (Figure 1A). These seizures were also detected behaviorally, reaching stage 2 or 3 on a modified Racine scale, characterized by forelimb clonus (Kim et al., 1999) and were thereby classified as ncSZ (Figure 1B). ncSZ were associated with aberrant EEG patterns, showing high intensity individual spikes (Figure 1A). Administration of 20 mM KA resulted in strong seizures characterized by frequent spike-bursts (Figure 1A), reaching Racine stage 4–5 and were thereby classified as cSZ (Figure 1B). As seizures induced by 20 mM KA lasted longer than 5 min, these seizures were also classified as convulsive status epilepticus (cSE). Twenty eight days later, ncSZ had not induced detectable granule cell dispersion and only regional astrogliosis in the hippocampus (Figure 1C–F). In contrast, administration of 20 mM KA resulted in detectable granule cell dispersion and severe astrogliosis (Figure 1C–F). The effects of the seizures were limited to the ipsilateral hemisphere (Figure 1C,E, quantifications not shown). As seizures induced by 0.74 mM and 2.22 mM did not induce SE, one important factor discriminating ncSZ from cSZ in addition to seizure intensity in this manuscript may be the presence of isolated vs. generalized seizures. These results show that granule cell dispersion and astrogliosis induced by intrahippocampal injection of KA in the DG depend on the intensity, as assessed by EEG and a modified Racine scale, of the generated seizures indicating that they may represent meaningful cellular parameters to distinguish cSZ from ncSZ.

### Seizure Intensity Conditions the Presence of Ectopic Immature Neurons in the DG

**FIGURE 2:**
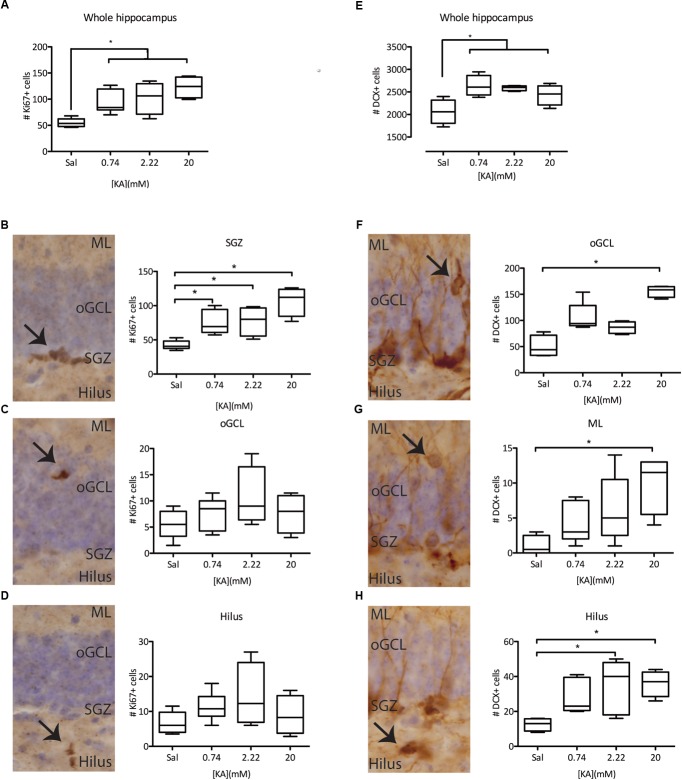
Characterization of KA dose-dependency on proliferation and immature (DCX+) neurons. **(A)** Immunohistochemistry against Ki67 reveals an induction in the total proliferation in the whole hippocampus of the dentate gyrus, independent of KA dose. This effect is mainly driven by increased proliferation in the SGZ **(B)** of the DG, and not by ectopic proliferation in the oGCL **(C)** or the Hilus **(D)**. **(E)** Immunohistochemistry against DCX reveals an induction of DCX+ immature neurons in the whole hippocampus, irrespective of the KA dose. However, ectopic DCX+ immature neurons are only present in the oGCL **(F)**, the ML **(G)**, or the hilus **(H)** after higher KA doses. ^∗^*p* < 0.05 arrows indicate immunopositive cells at each studied location.

We next asked if NSPC proliferation was affected by seizure intensity. All the KA doses tested significantly increased proliferation in the hippocampus 3 days after administration, as measured by the expression of Ki67 (Figure 2A). This increase in proliferation was accounted by a significant increase in proliferation in the subgranular zone (SGZ), with no detectable effect on proliferation in the outer granule cell layer (oGCL) or the Hilus (Figure 2B–D). To understand the possible long-term effects of increased NSPC proliferation, we characterized the numbers of (ectopic) immature neurons, characterized by the expression of doublecortin (DCX) in the oGCL, the molecular layer (ML), and the Hilus 28 days after KA administration. In line with the increased proliferation of NSPCs, the total number of DCX+ immature neurons in the hippocampus was also increased following all KA doses (Figure 2E). In contrast, the numbers of ectopic DCX^+^ immature neurons present in each one of the quantified areas did not change after 0.74 mM KA (Figure 2F–H). However, the administration of 2.22 mM KA significantly increased the numbers of ectopic immature neurons in the Hilus, but not in the oGCL or the ML (Figure 2F–H), while 20 mM KA was associated with the presence of ectopic immature neurons in all three areas tested (Figure 2F–H). These results indicate that the induction of proliferation and increase in the numbers of immature neurons are more general effects of seizure induction in the DG, while the presence of ectopic immature neurons, particularly in the oGCL and hilus, depends on seizure intensity and may distinguish cSZ from ncSZ.

### ncSZ Induces Neuroblast Proliferation in the DG

**FIGURE 3:**
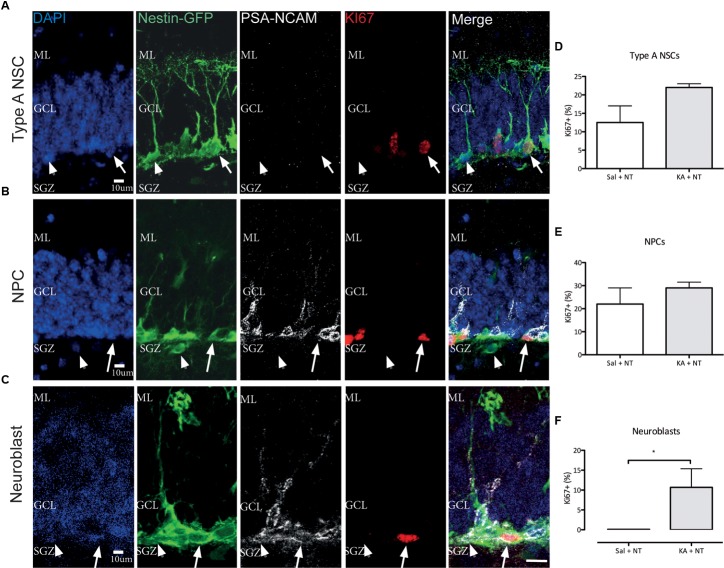
Effects of non-convulsive seizures on NSPC proliferation. Example confocal images of **(A)** Type A NSCs, **(B)** NPCs, and **(C)** Neuroblasts analyzed. **(D–F)** Quantification of Ki67+ proliferative cells among **(D)** Type A NSCs, **(E)** NPCs, and **(F)** Neuroblasts. The relative proliferative proportion of both Type A NSCs and NPCs did not change upon the induction of ncSZ, while a significant induction of proliferation was observed in neuroblasts. ^∗^*P* < 0.05.

Previous observations have demonstrated a complex cell type-specific proliferative response to cSZ in the SGZ engaging quiescent NSC and early neuroblast populations (Jessberger et al., 2005; Lugert et al., 2010). To characterize the proliferative response to ncSZ, we focused on three main proliferative cell types present in Nestin-GFP mice, a well-characterized NSPC reporter model system (Mignone et al., 2004; Encinas et al., 2011) (Figure 3B). These were: (1) activated NSCs (aNSC), identified by co-expression of Nestin-GFP and Ki67, the absence of PSA-NCAM and the presence of a triangular soma in the SGZ and a radial process orientated perpendicular to the GCL (Figure 3A); (2) proliferating neural progenitor cells (pNPC), identified by the expression of Nestin-GFP and Ki67, the absence of PSA-NCAM, the presence of polygonal soma in the SGZ and the lack of radial processes (Figure 3B); and (3) proliferating early neuroblasts, identified by co-expression of Nestin-GFP, KI67, and PSA-NCAM and the presence of polygonal soma in the SGZ (Figure 3C). We observed a significant increase in the numbers of proliferating neuroblasts and no differences in the numbers of aNSCs or pNPCs 3 days after the induction of ncSZ (Figure 3D–F). These results indicate that early neuroblast proliferation is specifically induced by ncSZ, as previously demonstrated for cSZ (Jessberger et al., 2005).

### ncSZ Induces Changes in the Composition of the NSC Pool in the DG

**FIGURE 4:**
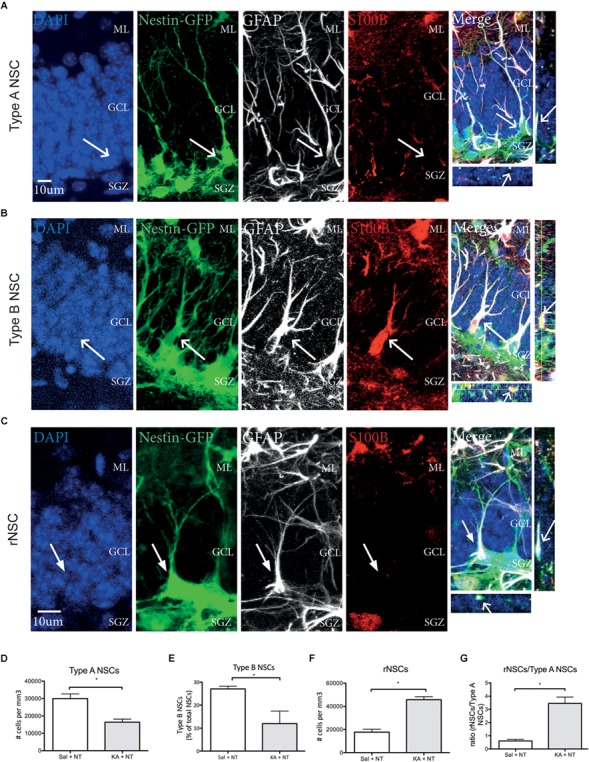
Effects of non-convulsive seizures on NSC identity. Example confocal images of **(A)** Type A NSCs **(B)** Type B NSCs **(C)** reactive NSCs, as assessed by marker expression and morphology. **(D)** Upon induction of ncSZ a significant loss of Type A NSCs was found. **(E)** At the same time, the relative proportion of Type B NSCs in the NSC pool also decreases upon induction of ncSZ. **(F)** Simultaneously, a significant increase in the number of rNSCs occurs, an effect that becomes even more visible when comparing the rNSC/Type A NSC ratio **(G)**. ^∗^*P* < 0.05.

Different NSC phenotypes have been identified in the DG based on marker expression and morphology (Kempermann et al., 2004; Sierra et al., 2015; Gebara et al., 2016). We first assessed the total numbers of Type A NSCs, detected as Nestin GFP+, GFAP+, S100β- cells with a triangular soma in the SGZ, a long radial process orientated perpendicular to the GCL and complex cellular processes reaching the ML (Kempermann et al., 2004) (Figure 4A); Type B NSC, detected as Nestin-GFP+, GFAP+, S100β+ cells with a triangular soma in the SGZ and a shorter radial process orientated perpendicular to the GCL (Gebara et al., 2016) (Figure 4B); and Reactive NSCs, detected as Nestin-GFP+, GFAP+ S100β- cells, with an enlarged soma located in the SGZ a thicker radial process orientated perpendicular to the GCL and less branched cellular processes not reaching the ML as compared to Type A NSCs (Sierra et al., 2015) (Figure 4C) and then asked if ncSZ may affect these three populations in the DG. Three days after induction ncSZ decreased the number of Type A NSCs (Figure 4D); decreased the number of Type B NSCs (Figure 4E) and increased the total number of reactive NSCs and the ratio of rNSCs/Type A NSCs (Figure 4F,G). As Type B and reactive NSC may both derive from Type A NSCs (Sierra et al., 2015; Gebara et al., 2016), these results indicate that ncSZ induces significant changes in the composition of the NSC pool in the DG, possibly promoting the generation of reactive NSCs at the expense of other NSC types. Although the generation of reactive NSCs at the expense of Type A NSCs has been documented after cSZ (Sierra et al., 2015), this is the first report of a possible effect of ncSZ on Type A and B NSCs.

### Co-administration of MiR-124 and -137 AMOs Prevents Neuroblast Proliferation in the DG Upon ncSZ

**FIGURE 5:**
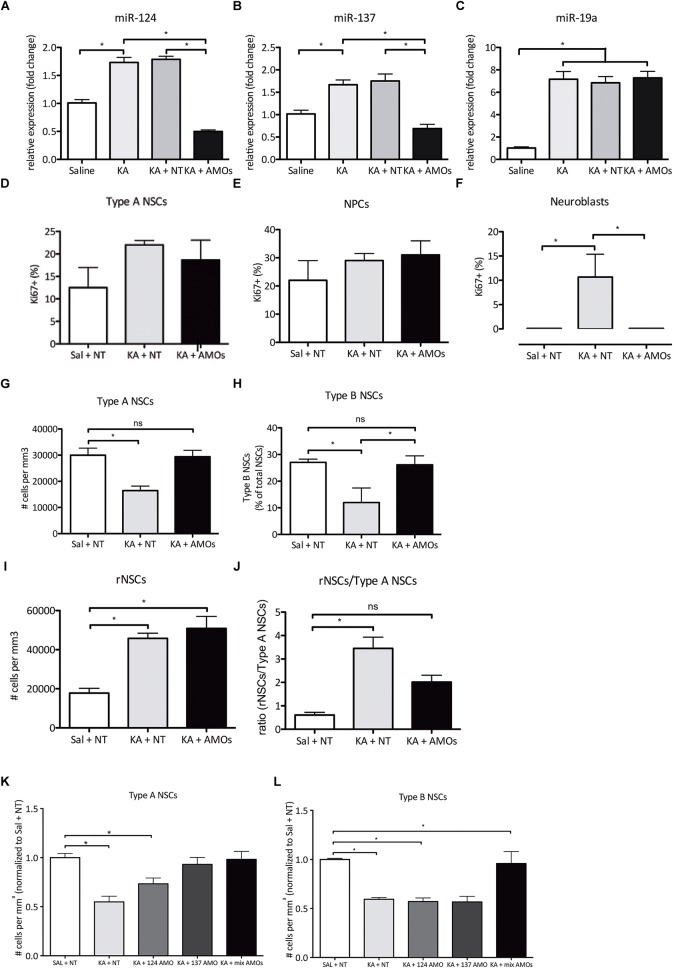
Combined treatment with anti miR-124 and -137 AMOs partially restores non-convulsive seizure-induced alterations in proliferation and NSC identity 3 days post SE onset. **(A)** Expression levels of miR-124 72 h post ncSZ and following AMO administration. **(B)** Expression levels of miR-137 72 h after ncSZ and following AMO administration. **(C)** Expression levels of the unrelated miR-19a 72 h after ncSZ, and following AMO administration. **(D–F)** combined AMO treatment successfully rescued the ncSZ-induced neuroblast proliferation, while not altering proliferation levels in Type A NSCs and NPCs. **(G)** The loss of Type A NSCs was rescued by the administration of the combined AMOs (Sal + NT vs. KA + AMOs, ns). **(H)** The decrease in the relative proportion of Type B NSCs among the NSC pool was also rescued by the AMO treatment (Sal + NT vs. KA + AMO, ns; KA + NT vs. KA + AMO, *P* < 0.05). **(I)** The induction of rNSCs upon ncSZ was not rescued by the AMO treatment; however, **(J)** as the number of Type A NSCs was restored, the rNSC/Type A NSC ratio was partially restored (Sal + NT vs. KA + AMO, ns). **(K)** Administration of individual AMOs 2 h post ncSZ partially prevents the loss of Type A NSCs. **(L)** Administration of individual AMOs 2 h post ncSZ did not prevent the loss of Type B NSCs. ^∗^*P* < 0.05.

Previous observations indicate that miR-124 and -137 are upregulated after seizures in the DG and their synergistic action contributes to the regulation of NSPCs (Schouten et al., 2015, 2016). First, we confirmed that miR-124 and miR-137 are upregulated in the DG 72 h after intrahippocampal KA-induced ncSZ (Figure 5A,B). To address possible effects of both microRNAs after ncSZ, we administered an equimolar combination of miRNA-124 and -137, or non-targeting (NT) AMOs as experimental control, to the DG, 2 h after the induction of ncSZ. Treatment with AMOs against microRNA-124 and -137 successfully decreased expression levels of both miRs 3 days post seizure induction, without affecting the expression levels of an unrelated miR (miR-19a) (Figure 5A–C), which has been used previously as a control (Reschke et al., 2017). Furthermore, NT control AMOs did not affect the expression of miR-124 or -137 (Figure 5A,B), supporting the specificity of the AMOs used. Treatment with this equimolar combination of specific miRNA-124 and -137 AMOs significantly inhibited the neuroblast-specific proliferative response observed after ncSZ, without significant effects on the numbers of proliferating NSCs or NPCs in the DG (Figure 5D–F).

### Co-administration of MiR-124 and -137 AMOs Prevents Changes in the Composition of the NSC Pool in the DG Upon ncSZ

Next, we asked if the co-administration of miR-124 and -137 AMOs could modify the NSC response to ncSZ in the DG. Again, we administered to the DG an equimolar combination of miRNA-124 and -137 or NT AMOs as experimental control, 2 h after the induction of ncSZ. We found that treatment with the miRNA-124 and -137 AMO combination significantly reduced the loss of Type A and Type B NSCs observed after ncSZ (Figure 5G,H). Finally, administration of the miRNA-124 and -137 AMO combination had no significant effect on the induction of reactive NSCs (Figure 5I). However, it significantly prevented the increase of the reactive NSC/Type 1 A NSC ratio induced by ncSZ (Figure 5J). To address whether these effects were linked to the synergistic effect of both miR-124 and miR-137, we then applied individual AMOs against either miR-137 or miR-124 2 h post seizure induction. To allow for comparison between the data obtained from the animals infused with the combined AMOs all data were normalized against controls (saline + NT AMOs). Administration of individual AMOs against miR-124 failed to rescue both the loss of Type A NSCs (Figure 5K) and Type B NSCs (Figure 5L), while individual AMOs against miR-137 prevented the loss of Type A NSCs (Figure 5K), but not the loss of Type B NSCs (Figure 5L). This indicates that the synergistic inhibition of both microRNAs plays a significant role in the preservation of NSCs after KA-induced ncSZ.

## Discussion

We show that intrahippocampal administration of increasing doses of KA results in a gradual increase in seizure intensity, induction of astrogliosis and ectopic immature neurons. In particular, ncSZ specifically induced proliferation of neuroblasts, increased the numbers of reactive NSC and resulted in a loss of Type A and Type B NSCs, thereby significantly altering the composition of the hippocampal NSC pool. Furthermore, we provide first evidence that a combination of specific AMOs directed against miR-124 and miR-137 administered locally to the DG shortly after the induction of ncSZ reverted the induction of neuroblast proliferation and the loss of Type A and Type B NSCs associated with ncSZ.

Most studies addressing the effects of seizures on the hippocampal NSC pool have used cSZ triggered by systemically administered chemoconvulsants like KA or pilocarpine (Parent et al., 1997; Bouilleret et al., 1999; Parent et al., 2006; Jessberger et al., 2007; Sibbe et al., 2012; Miltiadous et al., 2013; Cho et al., 2015; Twele et al., 2015). This may represent a limitation in the translational power of these studies, since not all epilepsy patients experience cSZ (Labovitz et al., 2001; Korff and Nordli, 2007; Rosenow et al., 2007). Systemically applied KA is an extensively used model with unquestionable value (Lévesque and Avoli, 2013). However, it triggers an all-or-nothing response that is inherently uncontrollable and may result in several indirect effects that complicate a correct interpretation of the earliest changes induced in brain tissue (Kienzler-Norwood et al., 2017). We have recently adapted an experimental protocol that allows for the local injection of chemoconvulsants in the DG, thereby providing an opportunity to elicit ncSZ in mice (Schouten et al., 2015; Sierra et al., 2015; Abiega et al., 2016; Bielefeld et al., 2017), as it has been also shown in guinea pigs (Carriero et al., 2012). An added advantage of the intrahippocampal injection of KA at lower doses may be the absence of GCL dispersion we report here. GCL dispersion has been observed frequently in mice and human, while it has seldom been shown in rat models of epilepsy (Pirttilä et al., 2005), making it a complicating feature present in some cSZ mice models. Using this extensively validated protocol, we here show that some of the effects of ncSZ on NSPC diverge from those of cSZ. Theoretically, if the lowest dose of KA that we injected was saturating the cellular response, then the local effects of higher KA doses may not be different. However, our data show at least some of the cellular parameters that we studied were differentially affected by the increasing KA doses, indicating that seizure intensity is a meaningful parameter in the study of the regulation of AHN by epileptic seizures. We show for the first time that ncSZ induces proliferation of early neuroblasts, similarly to cSZ (Jessberger et al., 2005). In contrast to observations done after cSZ (Lugert et al., 2010; Sierra et al., 2015), we show that ncSZ did not induce significant differences in the proliferation of Type A NSCs. Thus, our results suggest that Type A NSCs may only respond to high seizure intensities, and therefore depletion of the hippocampal NSC pool after ncSZ could be less severe than anticipated from the use of cSZ. This could have clinical implications since a significant number of epileptic patients experience ncSZ (Drislane, 2000; Alroughani et al., 2009).

Under normal conditions, NSCs in the adult hippocampus undergo asymmetric divisions that generate both NPCs and NSCs. This mechanism favors a neuronal progeny, while slowing down the depletion of the NSC pool (Encinas et al., 2011). The neuronal hyperactivity associated with cSZ promotes a switch toward symmetric division. This switch in division mode generates reactive NSCs, thereby depleting the NSC pool and impairing AHN (Sierra et al., 2015). Here we report that ncSZ also induced a significant increase in the number of reactive NSCs in the DG. Interestingly, reactive NSCs may also contribute to astrogliosis, that is commonly observed in epilepsy models and mTLE patients (Robel et al., 2015; Sierra et al., 2015). In agreement with this, the increase in reactive NSCs shortly after ncSZ correlated with the extent of astrogliosis 28 days after seizure onset. However, further experiments are required to definitively demonstrate how reactive NSCs may contribute to hippocampal astrogliosis after ncSZ.

Further, we provide evidence supporting the conclusion that ncSZ affects NSC composition in the DG. ncSZ increased the numbers of reactive NSCs, while simultaneously decreasing the number of Type A and B NSCs. Type B NSCs are derived from Type A NSCs by asymmetric division under physiological conditions (Gebara et al., 2016), while reactive NSCs are derived from Type A NSCs by symmetric division after cSZ (Sierra et al., 2015). We here show for the first time that ncSZ leads to a decrease in the number of Type B NSCs in the DG, which may result from a loss of Type A NSCs, or through direct depletion of Type B NSCs. Our observations support the idea that ncSZ may affect the division mode of Type A NSCs without increasing their total proliferation rate, in contrast to what has been observed before after cSZ (Lugert et al., 2010). However, an alternative explanation for our observations is that seizures prevent the generation of Type B from Type A NSC through hitherto unidentified mechanisms that do not involve a change in their division mode. The validation of either of these hypotheses requires further experimental evidence.

Several miRNAs regulate multiple steps in AHN, and the expression profiles of some of these miRNAs are altered in the DG upon seizure induction [reviewed in (Schouten et al., 2012; Bielefeld et al., 2016)]. Adding an extra level of complexity to miRNA-mediated regulation, a synergistic action between multiple miRNAs may function to enforce and stabilize gene-regulatory networks converging on biological functions or pathways that determine the fate of adult hippocampal NSCs (Pons-Espinal et al., 2017). In particular, synergy between mir-124 and -137 in NSCs controls a significant number of common targets involved in neurogenesis (Santos et al., 2016). Interestingly, miR-124 and -137 may regulate the division mode of NSCs (Gaiano and Fishell, 2002; Egger et al., 2010). Based on these previous observations, we explored the synergistic actions of miR-124 and -137 in NSC after ncSZ. AMOs (Weiler et al., 2006; Lennox et al., 2013), specifically inhibit miRNA actions *in vivo* and have shown therapeutic potential in treating epilepsy and concomitant cellular alterations in the hippocampus (Reschke et al., 2017; Beamer et al., 2018). We used a combination of AMOs targeting mir-124 and -137 administered locally to the DG to study possible cooperative functions. Strikingly, no proliferative neuroblasts were found 3 days after the onset of ncSZ in the combined AMO groups, indicating a crucial role for miR-124 and -137 synergy in the regulation of neuroblast proliferation upon ncSZ. Furthermore, the AMO treatment restored the numbers of Type A and Type B NSCs. Although the absolute number of reactive NSCs observed after ncSZ was unaffected by the AMO treatment, the reactive NSC/Type A NSC ratio was restored, indicating that prevention of Type A NSC loss is a main effect of the combined inhibition of miR-124 and miR-137 in the DG after ncSZ. To validate the functional role of microRNA cooperativity we administered individual AMOs against miR-124 or miR-137 2 h post ncSZ. Individual AMOs against miR-137 successfully prevented the loss of Type A NSCs, but not type B NSCs, while individual AMOs against miR-124 did not manage to prevent the loss of either type of NSCs. Since the combined administration of both AMOs did rescue the loss of Type B NSCs, these results indicate an action mediated by microRNA synergy, confirming the hypothesis that microRNA-124 and -137 act together to regulate many genes involved in the maintenance of NSCs after ncSZ.

## Materials and Methods

### Animals

Six week-old male Nestin-GFP^+/-^ mice (Mignone et al., 2004), were used in all experiments. Mice were housed in groups for 1 week prior to the start of the experiment under a 12-h light/dark cycle (lights on at 08.00) in a temperature- and humidity controlled room, with *ad libitum* access to food and water. All experiments were approved by the committee of animal health and care, University of Amsterdam (DED protocol #296 and #314, CCD 4925) and were performed in accordance with the guidelines and regulations of the European Union for the use of animals for scientific purposes. All mice were randomly assigned to experimental groups.

### Intrahippocampal KA and AMO Infusions

At post-natal day 42, mice were anesthetized in an airtight container using 5% isoflurane and placed in a stereotaxic apparatus. Anesthesia was maintained using 2% isoflurane during surgery. The intrahippocampal injection of KA was performed as described before (Bielefeld et al., 2017). In short, a small hole was drilled in the skull above both the hippocampi at the following coordinates: anteroposterior (AP) -2.0, mediolateral (ML) +1.5/-1.5. A pulled microcapillary was inserted and positioned at dorsoventral (DV) -2.0 and 50 nL of Saline (SAL) or KA (0.74, 2.22, or 20 mM) was infused into the DG using a microinjector (Nanoject II, Drummond Scientific). A second cohort of animals underwent the same stereotaxic procedure. Using the same bregma coordinates and a pulled microcapillary, 1.0 μL of an equimolar (50 μM) mix of microRNA-124 and -137 AMOs (Mirvana miRNA inhibitors, miRNA-124: CGUGUUCACAGCGGACCUUGAU; miRNA-137: ACGGGUAUUCUUGGGUGGAUAAU) was infused (50 μM, 0.2 μL/minute) using a microinjector (Nanoject II, Drummond Scientific). When necessary, mice were given intraperitoneal physiological saline injections to prevent dehydration after seizure onset.

### Electrode Implantation and EEG Recording

A separate cohort of mice underwent similar KA infusions and was additionally implemented with subdural goldplated stainless steel screw electrodes. The electrode implantation and EEG recordings were performed as described before (Schouten et al., 2015; Bielefeld et al., 2017). In short, electrodes were placed beneath the dura in the holes drilled for KA infusion. An additional dual reference and ground electrode was placed above the frontal cortex (AP–0.1, ML +0.1). All electrodes were attached to the skull using dental cement (Simplex Rapid, Kemdent), and attached to a wireless EEG recording system (Neurologger, TSE) allowing 72 h non-stop EEG acquisition.

### Seizure Classification

Seizures were scored using both EEG data and behavioral assessment based on a modified Racine scale (Racine, 1972; Schouten et al., 2015). We classified seizures to be non-convulsive based on behavioral assessment, with seizures not reaching higher than three on the Racine scale. Seizures reaching Racine scale 4 or higher were classified as cSZ. Seizures reaching Racine scale 4 or higher and lasting longer than 5 min were classified as cSE as in agreement with the official guidelines of the International League Against Epilepsy (Trinka et al., 2015). EEG data was analyzed separately, and both single spikes as well as spike bursts were manually identified.

### Tissue Collection

Seventy two hours or 28 days post KA infusion mice were sacrificed by transcardial perfusion with PBS followed by 4% paraformaldehyde (pH 7.4). Brains were extracted and stored overnight in paraformaldehyde at 4°C, followed by long-term storage in PBS containing 0.01% azide at 4°C. Serial 40 μm-thick coronal sections were obtained using a microtome (Jung). A second cohort of animals was sacrificed by decapitation 3 days post ncSZ and the DG was microdissected and snap frozen.

### Immunohistochemistry

All fluorescent immunohistochemical experiments were performed following a standard procedure. Sections were incubated with a blocking and permeabilization solution (PBS containing 0.3% Triton-100X and 2% serum) for 30 min at room temperature, followed by incubation with primary antibodies in the same solution for 1 h at room temperature followed by overnight incubation at 4°C. After thorough washing with PBS, sections were incubated with fluorochrome-conjugated secondary antibodies for 2 h at room temperature. All sections were again thoroughly washed in PBS and PB and subsequently mounted on slides. Slides were enclosed using vectashield containing DAPI and dried. The following antibodies were used Chicken anti GFP, mouse anti GFAP, mouse anti PSA-NCAM, Rabbit anti S100B, rabbit anti Ki67.

DAB-based immunohistochemistry was performed as described previously (Oomen et al., 2010; Schouten et al., 2015) using the following antibodies: goat anti DCX (Santa Cruz, 1:500), rabbit anti Ki67 (Abcam, 1:500), mouse anti GFAP (Millipore, 1:1000). Nissl staining was performed using Cresyl violet, as previously described (Heinrich et al., 2006).

### Image Acquisition and Quantification

All fluorescent images were acquired using a Zeiss LSM 510 confocal microscope and the corresponding manufacturer software. From each animal, every eighth serial coronal slice containing the dorsal hippocampus was analyzed. Per slice, four 30 μM-thick z-stack images of the dorsal hippocampus were obtained equally distributed along the suprapyramidal and infrapyramidal blade of the DG using a 40× magnification and transferred into ImageJ software for editing and analysis.

Quantitative analysis of cell populations was performed by manual counting of cell populations based on the coexpression of cell-type specific markers as described in the results section and corrected for the analyzed volume of the measured area. Images of all DAB-based immunohistochemistry were acquired using a Nikon Eclipse fluorescence microscope and transferred to ImageJ for analysis. Ki67+ cells were counted manually and assigned to one of the following locations: SGZ, outer GCL, or the Hilus. Per hippocampus, eight coronal, 40 μm thick sections were sampled along the rostro-caudal axis (corresponding with Bregma: -1.34, -1.70, -2.06, -2.46, -2.92, -3.16, -3.52, -3.80) as described before (Abbink et al., 2017).

Total DCX+ cells were assessed using a stereological approach as described before (Oomen et al., 2010; Schouten et al., 2015). Ectopic DCX+ cells were counted manually and assigned to one of three anatomical locations: outer GCL, ML, or Hilus. The SGZ was defined as two cell bodies distance from the GCL, while the outer GCL was here defined as the outer half of the GCL. Gliosis was analyzed by measuring GFAP surface area coverage in the whole hippocampus using ImageJ. GCL dispersion was assessed by measuring the total thickness of the GCL using ImageJ.

### microRNA Expression Analysis

RNA was isolated from snap frozen fresh micro dissected DG tissue and subsequently transcribed into cDNA using a taqman microRNA reverse transcription kit combined with specific miR- reverse transcription primers. MicroRNA expression levels were determined using taqman microRNA assays specific for miR-124 (assay ID:001182), miR-137 (assay ID:001129), and miR-19a (assay ID:002544) and levels were normalized to RNU6b (assay ID:001093) as described before (Schouten et al., 2015).

### Statistical Analysis

All statistical analyses were carried out using Graphpad Prism 5.0. All data are shown as mean +/- SEM and *p* < 0.05 was considered significant. All comparisons were tested using a 1-way ANOVA and Tukey *post hoc* analysis, unless specifically stated otherwise.

## Author Contributions

PB, MS, GM, MB, KG, SK, AT, AV, RW, and DW performed experiments and analyzed data. PL, JE, and CF participated in experimental design, result discussion, interpretation, and manuscript preparation. PB and CF conceived the study, designed experiments, analyzed and interpreted results, and wrote the manuscript.

## Conflict of Interest Statement

The authors declare that the research was conducted in the absence of any commercial or financial relationships that could be construed as a potential conflict of interest.
